# A Novel microRNA and transcription factor mediated regulatory network in schizophrenia

**DOI:** 10.1186/1752-0509-4-10

**Published:** 2010-02-15

**Authors:** An-Yuan Guo, Jingchun Sun, Peilin Jia, Zhongming Zhao

**Affiliations:** 1 Department of Biomedical Informatics, Vanderbilt University School of Medicine, Nashville, TN, USA; 2 Department of Psychiatry, Vanderbilt University School of Medicine, Nashville, TN, USA; 3 Bioinformatics Resource Center, Vanderbilt-Ingram Cancer Center, Vanderbilt University, Nashville, TN, USA

## Abstract

**Background:**

Schizophrenia is a complex brain disorder with molecular mechanisms that have yet to be elucidated. Previous studies have suggested that changes in gene expression may play an important role in the etiology of schizophrenia, and that microRNAs (miRNAs) and transcription factors (TFs) are primary regulators of this gene expression. So far, several miRNA-TF mediated regulatory modules have been verified. We hypothesized that miRNAs and TFs might play combinatory regulatory roles for schizophrenia genes and, thus, explored miRNA-TF regulatory networks in schizophrenia.

**Results:**

We identified 32 feed-forward loops (FFLs) among our compiled schizophrenia-related miRNAs, TFs and genes. Our evaluation revealed that these observed FFLs were significantly enriched in schizophrenia genes. By converging the FFLs and mutual feedback loops, we constructed a novel miRNA-TF regulatory network for schizophrenia. Our analysis revealed EGR3 and hsa-miR-195 were core regulators in this regulatory network. We next proposed a model highlighting EGR3 and miRNAs involved in signaling pathways and regulatory networks in the nervous system. Finally, we suggested several single nucleotide polymorphisms (SNPs) located on miRNAs, their target sites, and TFBSs, which may have an effect in schizophrenia gene regulation.

**Conclusions:**

This study provides many insights on the regulatory mechanisms of genes involved in schizophrenia. It represents the first investigation of a miRNA-TF regulatory network for a complex disease, as demonstrated in schizophrenia.

## Background

Schizophrenia is a complex, chronic and severe brain disorder. So far, its pathophysiology and molecular mechanisms have remained poorly understood [1]. In the last decade numerous linkage and association studies, including a few genome-wide association studies (GWAS), have been performed to identify genetic predispositions to the disease, but most studies have been inconclusive. The limited success in the detection of genetic factors led us to hypothesize that schizophrenia is likely caused by the altered expression of many genes, which may individually contribute only a small risk, but may in aggregate interact at the biological pathway or gene-network level.

Recently, patterns of differential gene expression have been identified between schizophrenia case and control samples [2,3]. MicroRNAs (miRNAs) and transcription factors (TFs) are main regulators of gene expression. MiRNAs are short endogenous noncoding RNAs that mediate post transcriptional regulation and regulate a wide range of biological processes and diseases [4,5]. In the nervous system, studies have reported involvement of miRNAs in brain development, neuronal differentiation, and synaptic plasticity, all processes that have been implicated in neurological syndromes such as schizophrenia, fragile × syndromes, Parkinson's disease and Huntington's disease [5]. Specifically, 18 miRNAs were recently found to be differentially expressed in post-mortem brain samples of schizophrenia patients and controls [6,7]. Interestingly, a case-control association study revealed that two single nucleotide polymorphisms (SNPs) in miRNAs hsa-miR-206 and hsa-miR-198 were significantly associated with schizophrenia [8]. Furthermore, brain miRNAs affected by a microdeletion syntenic to human 22q11.2 were found in mouse models and human individuals carrying this microdeletion are at high risk of developing schizophrenia [9]. It has also been reported that miRNA hsa-miR-195 regulates *BDNF *and alters the expression of downstream GABAergic transcripts in schizophrenia [10]. Most recently, studies found that a miRNA regulates signaling downstream from the NMDA receptor, suggesting miRNAs as a new mechanism for altering brain gene expression in schizophrenia [11,12]. This accumulating data suggests that miRNAs may play important roles in the expression of genes linked to schizophrenia.

Transcription factors are essential regulators of gene expression in all living organisms. A TF regulates transcription of its target gene by specifically binding to the transcription factor binding site (TFBS) in the gene's promoter region. Since expression of an miRNA may be regulated by a TF [13], TF and miRNA may reciprocally regulate one another to form feedback loops, or alternatively, both TF and miRNA may regulate their target genes and form feed-forward loops (FFLs). Two recent studies explored hundreds of potential miRNA-mediated feedback and feed-forward loops at the genome level in mammals and found some interesting regulatory motifs [14,15]. Besides, Martinez *et al*. [16] combined experimental and computational methods and identified 23 miRNA-TF composite feedback loops in *C. elegans*. Several feedback loops and FFLs have been experimentally verified in mammals, such as feedback loops between ZEB1/SIP1 and miR-200 family in embryological development, E2Fs and miR-17/20 in cellular apoptosis, PITX3 and miR-133b in midbrain dopamine neurons, and a FFL E2Fs-Myc-miR-17/20 [17-19]. These studies were performed at the whole genome level by a computational approach or for specific FFLs by experimental validation, rather than a comprehensive miRNA-mediated network analysis for a specific complex disease or tissue.

In this study, we explored miRNA-TF regulatory networks in schizophrenia. Among schizophrenia candidate genes (SZGenes), we identified the potential targets of TFs and schizophrenia related miRNAs. These datasets and their regulations were used for miRNA-mediated feedback and feed-forward loop analysis. We revealed some schizophrenia related miRNA-TF regulatory modules and constructed a converged miRNA-TF regulatory network in which EGR3 and hsa-miR-195 served as core regulators. By combining miRNA-TF network analysis and literature survey, we proposed a pathway model highlighting EGR3 and miRNAs involving in the signal transduction and regulatory pathways in schizophrenia.

## Results

### miRNAs and TFBSs

**Figure 1:**
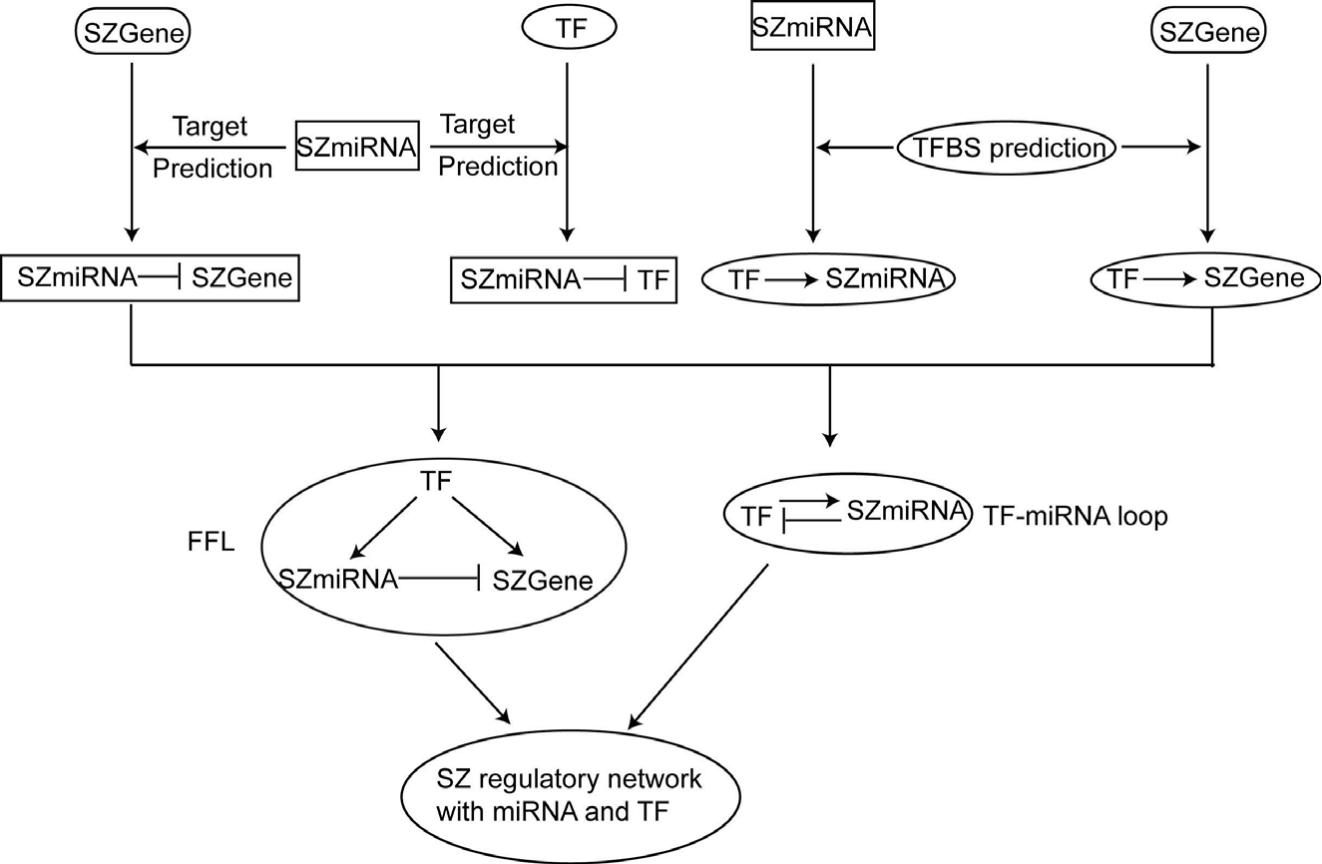
**An overview of miRNAs, TFBSs/TFs and their regulatory network in schizophrenia**. SZ: schizophrenia; FFL: feed-forward loop.

Our goal is to explore miRNA and TF regulation in schizophrenia genes. Figure 1 provides an overview of miRNA and TF mediated regulatory network construction. We first compiled a list of 20 experimentally verified schizophrenia related miRNAs (SZmiRNAs), which matched 21 mature miRNAs and 29 miRNA precursors (Table 1). Most of the 29 SZmiRNAs are conserved in vertebrate genomes and 9 are even conserved in *Drosophila*. Only one (hsa-miR-198) is primate-specific and two (hsa-miR-195 and hsa-miR-206) are mammal-specific. Sixteen SZmiRNAs (55%) were found in miRNA clusters. For comparison, we also collected and curated 87 brain expressed and 79 non-brain expressed mature miRNAs, which corresponded to 105 and 94 miRNA precursors, respectively (see Additional file 1).

**Table 1:** Location and conservation information of schizophrenia related miRNAs

miRNA		Location (Chr: start-end [strand])	Host gene^a^	Taxonomy conservation^b^
hsa-let-7g		3: 52277334-52277417 [-]	*WDR82*	V
hsa-miR-106b		7: 99529552-99529633 [-]	*MCM7*	V
hsa-miR-181b	hsa-miR-181b-1	1: 197094625-197094734 [-]	Intergenic	V
	hsa-miR-181b-2	9: 126495810-126495898 [+]	*NR6A1 *(antisense)	V
hsa-miR-195		17: 6861658-6861744 [-]	Intergenic	M
hsa-miR-198		3: 121597205-121597266 [-]	*FSTL1 *(3'UTR)	P
hsa-miR-206		6: 52117106-52117191 [+]	Intergenic	M
hsa-miR-20b		X: 133131505-133131573 [-]	Intergenic	V
hsa-miR-212		17: 1900315-1900424 [-]	Intergenic	V
hsa-miR-24	hsa-miR-24-1	9: 96888124-96888191 [+]	*C9orf3*	V
	hsa-miR-24-2	19: 13808101-13808173 [-]	Intergenic	V
hsa-miR-26b		2: 218975613-218975689 [+]	*CTDSP1*	V
hsa-miR-29a		7: 130212046-130212109 [-]	*AP4M1 *(antisense)	V
hsa-miR-29b	hsa-miR-29b-1	7: 130212758-130212838 [-]	*AP4M1 *(antisense)	V
	hsa-miR-29b-2	1: 206042411-206042491 [-]	Intergenic	V
hsa-miR-29c		1: 206041820-206041907 [-]	Intergenic	V
hsa-miR-30a		6: 72169975-72170045 [-]	*C6orf155*	V
hsa-miR-30b		8: 135881945-135882032 [-]	Intergenic	V
hsa-miR-30d		8: 135886301-135886370 [-]	Intergenic	V
hsa-miR-30e		1: 40992614-40992705 [+]	*NFYC*	V
hsa-miR-7	hsa-miR-7-1	9: 85774483-85774592 [-]	*HNRNPK*	A
	hsa-miR-7-2	15: 86956060-86956169 [+]	Intergenic	A
	hsa-miR-7-3	19: 4721682-4721791 [+]	*C19orf30*	A
hsa-miR-9	hsa-miR-9-1	1: 154656757-154656845 [-]	*C1orf61*	A
	hsa-miR-9-2	5: 87998427-87998513 [-]	Intergenic	A
	hsa-miR-9-3	15: 87712252-87712341 [+]	Intergenic	A
hsa-miR-92a	hsa-miR-92a-1	13: 90801569-90801646 [+]	Intergenic	A
	hsa-miR-92a-2	X: 133131234-133131308 [-]	Intergenic	A
hsa-miR-92b		1: 153431592-153431687 [+]	Intergenic	A

^a^Antisense: miRNA and its host gene are on opposite strand. 3'UTR: miRNA locating on the 3'UTR of its host gene. The remaining miRNAs are in the intron of their host genes.

^b^Taxonomy conservation: P: primates (human, chimp, rhesus monkey); M: mammals (human, mouse, rat, dog); V: vertebrates (human, mouse, rat, dog, chicken, frog, fish); A: animals (human, mouse, chicken, fish, fly).

**Figure 2:**
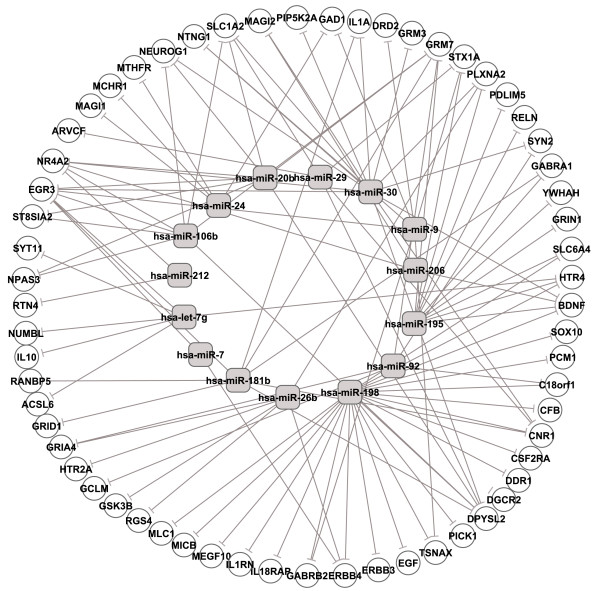
**Schizophrenia candidate genes targeted by schizophrenia related miRNAs**. To simply the figure, miRNA family names are used for their precursors.

We predicted miRNA targets in SZGenes by parsing TargetScan prediction results. Among the 160 SZGenes, 61 were potential targets of our complied 29 SZmiRNAs. Figure 2 displays these miRNA and target pairs. Among the 61 target genes, the top three genes targeted by the largest number of SZmiRNAs were *EGR3*, *DPYSL2 *and *CNR1*, which were targeted by 15, 13 and 11 SZmiRNAs, respectively. Among the 29 SZmiRNAs, the miRNAs targeting the largest number of SZGenes were hsa-miR-198, miRNAs in miR-30 family and hsa-miR-195, which targeted 23, 14 and 11 genes, respectively (see Additional file 2: Table S1). Hsa-miR-198 had the largest number of targets because it is a primate-specific miRNA and the predicted target sites may not be conserved with a high false positive rate.

To test whether we observed more SZmiRNA targets in the 160 SZGenes, we ran a permutation to count the number of targets of each SZmiRNA in 160 randomly selected genes, and repeated this process 10,000 times. Most (25 of 29, 86.2%) of SZmiRNAs had a significantly larger number of targets in SZGenes than randomly selected genes (t-test, *p*-value < 0.001), while hsa-miR-206 had fewer targets in SZGenes and the difference for 3 miRNAs in hsa-miR-7 family was not significant (see Additional file 2: Fig. S1).

Using stringent criteria (Z score >2.33 in UCSC Genome Browser) and conservation among the human, mouse and rat genomes, we obtained 517 TFBSs in the promoter regions of 115 of the 160 SZGenes and 184 TFBSs in the promoter regions of 18 of the 29 SZmiRNAs. Among the 115 SZGenes, 79 (68.7%) had fewer than 5 TFBSs and 10 (8.7%) had more than 10 TFBSs. Among the SZmiRNAs, hsa-miR-212 and hsa-miR-195 had more than 20 TFBSs (see Additional file 2: Fig. S2). These observations seemed to reflect a complex regulation of schizophrenia related genes, TFBSs and miRNAs.

### Feed-forward loops (FFLs) in schizophrenia

We obtained 32 FFLs when we combined the regulatory relationship of SZGenes, SZmiRNAs and TFBSs (Table 2). We performed following two tests to evaluate the enrichment of these observed FFLs in the SZGenes. First, we compared the FFLs obtained from SZmiRNAs with those from brain miRNAs or non-brain miRNAs and then evaluated the significance by Fisher's exact test. The difference was highly significant in the comparison of FFLs by SZmiRNAs versus non-brain miRNAs (*p *= 1.80 × 10^-5^) and significant by SZmiRNAs versus brain miRNAs (*p *= 0.02) using the same SZGenes (Table 3). To increase the confidence, we did similar FFL analysis using three other schizophrenia candidate gene lists (see Methods). When we compared SZmiRNAs with non-brain miRNAs, the *p*-value was always highly significant, indicating that we observed more FFLs by SZmiRNAs than by non-brain miRNAs (Table 3). We noticed that the *p*-values in the comparison between SZmiRNAs and brain miRNAs were slightly significant or even insignificant. This likely represents some brain miRNAs in our data set that may be schizophrenia related but that have not yet been reported.

**Table 2:** FFLs among TFBSs, SZmiRNAs and schizophrenia genes (SZGenes)

No.	SZGene	miRNA	TFBS matrix	Matrix consensus	TF symbol
1	*ARVCF*	hsa-miR-29b-2	V$TCF11MAFG_01	ATGACTCAGCANTTNNG	NFE2L1, MAFG
2	*ARVCF*	hsa-miR-29c	V$TCF11MAFG_01	ATGACTCAGCANTTNNG	NFE2L1, MAFG
3	*BDNF*	hsa-miR-195	V$NFKB_C	GGGACTTTCCA	NFKB1, NFKB2
4	*DRD2*	hsa-miR-9-3	V$RP58_01	AACATCTGGA	ZNF238
5	*EGR3*	hsa-miR-181b-1	V$IK3_01	TNYTGGGAATACC	IKZF1
6	*EGR3*	hsa-miR-20b	V$NGFIC_01	TGCGTRGGYGK	EGR1, EGR2, EGR3, EGR4
7	*EGR3*	hsa-miR-9-1	V$MZF1_02	KNNNKAGGGGNAA	MZF1
8	*EGR3*	hsa-miR-9-1	V$OCT_C	CTNATTTGCATAY	POU2F1; POU2F2
9	*EGR3*	hsa-miR-9-3	V$CREBP1_Q2	VGTGACGTMACN	CREB1, ATF2
10	*GAD1*	hsa-miR-9-2	V$OLF1_01	CDABTCCCYAGRGARBNKG	EBF1
11	*GAD1*	hsa-miR-9-3	V$NRSF_01	TTCAGCACCACGGACAGMGCC	REST
12	*GRIN1*	hsa-miR-195	V$EGR1_01, V$NGFIC_01	TGCGTRGGYGK	EGR1, EGR2, EGR3, EGR4
13	*GRM7*	hsa-miR-195	V$AHRARNT_01	KNNKNNTYGCGTGCMS	AHR, ARNT
14	*GRM7*	hsa-miR-195	V$NGFIC_01	TGCGTRGGYGK	EGR1, EGR2, EGR3, EGR4
15	*GRM7*	hsa-miR-20b	V$NGFIC_01	TGCGTRGGYGK	EGR1, EGR2, EGR3, EGR4
16	*GRM7*	hsa-miR-20b	V$TAL1BETAE47_01	AACAGATGKT	TCF3, TAL1
17	*GRM7*	hsa-miR-92a-2	V$NGFIC_01	TGCGTRGGYGK	EGR1, EGR2, EGR3, EGR4
18	*GRM7*	hsa-miR-92a-2	V$TAL1BETAE47_01	AACAGATGKT	TCF3, TAL1
19	*HTR4*	hsa-miR-195	V$AHRARNT_01	KNNKNNTYGCGTGCMS	AHR, ARNT
20	*HTR4*	hsa-miR-195	V$GCNF_01	TCAAGKTCAAGKTCA	NR6A1
21	*MTHFR*	hsa-miR-24-2	V$AHRARNT_02	KNNKNNTYGCGTGCMS	AHR, ARNT
22	*NEUROG1*	hsa-miR-20b	V$POU6F1_01	GCATAAWTTAT	POU6F1
23	*NR4A2*	hsa-miR-20b	V$PAX4_03	YCACCCB	PAX4
24	*NR4A2*	hsa-miR-212	V$BACH2_01	SRTGAGTCANC	BACH2
25	*NR4A2*	hsa-miR-212	V$CEBP_C	GWVTKNKGYAAKNSAYA	CEBPA
26	*NR4A2*	hsa-miR-212	V$CREB_01, V$CREBP1CJUN_01, V$CREBP1_Q2	TGACGTMA	CREB1, ATF2
27	*NR4A2*	hsa-miR-212	V$FREAC3_01	GTAAATAAACA	FOXC1
28	*NTNG1*	hsa-miR-9-3	V$PAX5_02	RRMSWGANWYCTNRAGCGKRACSRYNSM	PAX5
29	*PDLIM5*	hsa-miR-195	V$AHRARNT_01	KNNKNNTYGCGTGCMS	AHR, ARNT
30	*RTN4*	hsa-miR-212	V$BACH1_01, V$BACH2_01	SRTGAGTCA	BACH1, BACH2
31	*TSNAX*	hsa-miR-9-1	V$OCT_C	CTNATTTGCATAY	POU2F1, POU2F2
32	*YWHAH*	hsa-miR-195	V$EGR1_01	TGCGTRGGYGK	EGR1, EGR2, EGR3, EGR4

**Table 3:** Statistics of FFLs identified by miRNAs among four schizophrenia gene lists

miRNA dataset	No. of miRNAs	160 SZGenes		75 SZGenes		124 SZGenes		270 SZGenes	
					
		FFLs	*p*-value	FFLs	*p*-value	FFLs	*p*-value	FFLs	*p*-value
SZmiRNAs	29	32		12		27		38	
Brain miRNAs	105	55	0.020	26	0.204	49	0.035	87	0.120
Non-brain miRNAs	94	24	1.80 × 10^-5^	11	9.86 × 10^-3^	20	4.79 × 10^-5^	27	2.97 × 10^-7^

*P*-value was calculated by Fisher's exact test between SZmiRNAs and brain miRNAs or between SZmiRNAs and non-brain miRNAs.

Secondly, we ran 10,000 random simulations (see Methods). In each run, since there were 209 miRNA target pairs between SZmiRNAs and SZGenes, we randomly selected 209 miRNA target pairs out of all target pairs of the 29 SZmiRNAs and calculated the number of FFLs among TFBSs, SZmiRNAs and those randomly selected target genes. We calculated a *p *value = 0.0009, indicating that our observed FFLs differed significantly from chance.

### miRNA and TF regulatory network in schizophrenia

**Figure 3:**
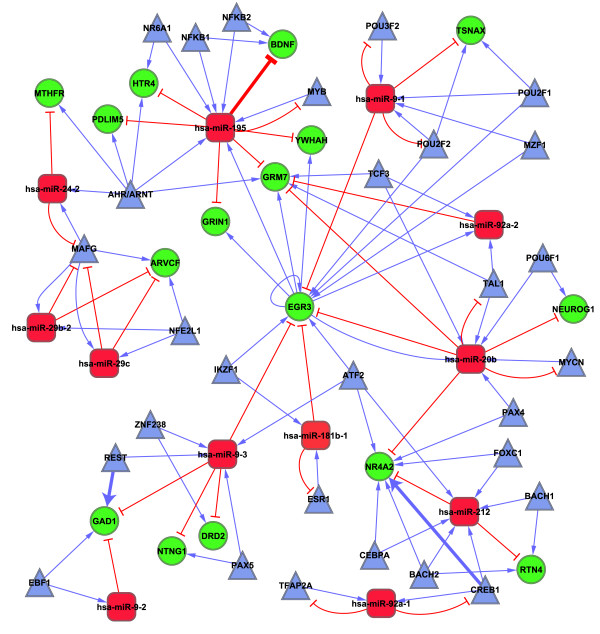
**A miRNA and TF mediated regulatory network in schizophrenia**. Red: schizophrenia related miRNAs; green: schizophrenia candidate genes; blue: TFs. Three thick lines denote regulations with experimental evidence.

TF and miRNA may regulate one another and form a composite feedback loop. We identified 14 SZmiRNA-TF mutual regulatory loops (pairs). Twelve of these had at least one TF or miRNA in the TF-SZmiRNA-SZGene FFLs and 5 pairs had all components included in the FFLs (see Additional file 2: Table S2). We merged the 12 FFL-related SZmiRNA-TF loops with TF-SZmiRNA-SZGene FFLs and constructed a miRNA-TF regulatory network for schizophrenia. It included 12 SZmiRNAs, 16 SZGenes, 29 TFs and 110 links (edges) between these molecules (nodes) (Figure 3). Among these 16 SZGenes, several (*DRD2*, *GRIN1*, *GRM7 *and *GAD1*) are related to three neurochemical hypotheses in the molecular mechanisms of schizophrenia, i.e., the dopamine, glutamatergic and GABAergic hypotheses [1]. Three TFs (ESR1, MYB and TFAP2A) in this network had association information in the SchizophreniaGene database [20] but only the *ESR1 *gene had a positive association study [21]. Moreover, there were 3 pairs of regulation (hsa-miR-195 represses *BDNF *gene, TF REST regulates *GAD1 *gene, and TF CREB1 regulates *NR4A2 *gene) that had been previously experimentally verified [10] or annotated in the Ingenuity Knowledge Base [22].

### Subnetworks for core genes in the miRNA-TF regulatory network

There were 5 core genes (*EGR3*, hsa-miR-195, hsa-miR-20b, hsa-miR-9-3 and *GRM7*) in the miRNA-TF regulatory network (Figure 3) according to the definition in the Methods section. We extracted subnetworks for these 5 core genes by including the core genes and their directly linked molecules in the miRNA-TF regulatory network (see Additional file 2: Fig. S3). In this subnetwork analysis, *EGR3 *stood out as a promising gene and regulator. As a gene, it is regulated by 5 TFs and 4 SZmiRNAs, while in its capacity as a TF, it regulates 3 SZGenes and 3 SZmiRNAs. *EGR3 *is the only gene in the network that links to all 4 of the other core genes.

Among the 3 core miRNAs (hsa-miR-195, hsa-miR-20b and hsa-miR-9-3), hsa-miR-195 seems most promising. It regulates 6 of the 16 SZGenes in the network, while it is also regulated by 6 TFs (Figure 3, see Additional file 2: Fig. S3B). We examined the predicted targets of these 3 miRNAs on all human genes and found 734, 725, and 826 predicted targets, respectively. We next examined the enriched pathways of these predicted targets of the 3 core miRNAs using the Ingenuity Pathway Analysis (IPA) tool. Interestingly, we found many neuron or schizophrenia related pathways, such as axonal guidance signaling and reelin signaling in neurons (Table 4). There were two enriched pathways shared by the targets of these 3 core miRNAs: axonal guidance signaling and Ephrin receptor signaling. Axon guidance is one of the critical steps in the proper formation of a neuronal network [23], and Ephrin/Ephrin receptor signaling is one of the bidirectional signaling systems implicated in the control of axon guidance and synapse formation in many neural systems [24]. This analysis suggests that these 3 core miRNAs may have important regulatory roles in the neuronal network. Finally, we examined the enriched GO terms of these predicted targets. Interestingly, among the enriched GO terms were several related to regulation and neurodevelopment such as "transcription regulation", "neuron differentiation" and "neurogenesis" (see Additional file 2: Table S3).

**Table 4:** Pathways enriched among the targets of 3 core miRNAs

miRNA	Enriched pathway of miRNA targets^a^	*p*-value
hsa-miR-195	TGF-β signaling	3.98 × 10^-5^
	*Axonal guidance signaling*	1.35 × 10^-4^
	Wnt/β-catenin signaling	2.04 × 10^-4^
	BMP signaling pathway	3.47 × 10^-4^
	FGF signaling	7.59 × 10^-4^
	Protein ubiquitination pathway	9.12 × 10^-4^
	CDK5 signaling	1.55 × 10^-3^
	PTEN signaling	1.86 × 10^-3^
	Amyloid processing	2.00 × 10^-3^
	B cell receptor signaling	3.72 × 10^-3^
	Inositol phosphate metabolism	4.57 × 10^-3^
	*PI3K/AKT signaling*	4.79 × 10^-3^
	*Reelin signaling in neurons*	4.79 × 10^-3^
	*Synaptic long term potentiation*	7.08 × 10^-3^
	PPARα/RXRα activation	7.59 × 10^-3^
	*Ephrin receptor signaling*	7.59 × 10^-3^
	Insulin receptor signaling	9.12 × 10^-3^
hsa-miR-20b	Cell cycle: G1/S checkpoint regulation	1.10 × 10^-4^
	*Reelin signaling in neurons*	2.75 × 10^-4^
	B cell receptor signaling	3.55 × 10^-4^
	*Axonal guidance signaling*	4.17 × 10^-4^
	TGF-β signaling	5.50 × 10^-4^
	p53 signaling	1.12 × 10^-3^
	*Semaphorin signaling in neurons*	1.29 × 10^-3^
	SAPK/JNK signaling	1.35 × 10^-3^
	Hypoxia signaling in the cardiovascular system	2.04 × 10^-3^
	Wnt/β-catenin signaling	3.24 × 10^-3^
	*PI3K/AKT signaling*	3.89 × 10^-3^
	*CNTF signaling*	3.98 × 10^-3^
	Cell cycle: G2/M DNA damage checkpoint regulation	5.75 × 10^-3^
	Circadian rhythm signaling	6.03 × 10^-3^
	*Ephrin receptor signaling*	6.03 × 10^-3^
	Factors promoting cardiogenesis in vertebrates	7.41 × 10^-3^
	FGF signaling	7.41 × 10^-3^
	HIF1α signaling	7.59 × 10^-3^
	*ERK/MAPK signaling*	8.71 × 10^-3^
hsa-miR-9-3	*ERK/MAPK signaling*	1.58 × 10^-4^
	Regulation of actin-based motility by Rho	2.88 × 10^-3^
	Clathrin-mediated endocytosis	3.72 × 10^-3^
	*Axonal guidance signaling*	3.89 × 10^-3^
	*Ephrin receptor signaling*	4.17 × 10^-3^
	Fcγ receptor-mediated phagocytosis in macrophages and monocytes	6.31 × 10^-3^

^a^Pathways in italic are related to nervous system or schizophrenia.

### SNPs on miRNA target sites, TFBSs, and miRNA genes

SNPs on miRNA target sites and TFBSs have been associated with many complex diseases [4,25,26]. So far, most of the SNPs associated with schizophrenia have not been in exonic regions [20]. Thus, it is important to examine SNPs in these schizophrenia related miRNA genes and their target sites and TFBSs. We identified 7 SNPs on the SZmiRNA target sites of 7 SZGenes, 14 SNPs on TFBSs of 13 SZGenes and 5 SNPs on TFBSs of 5 SZmiRNAs. Moreover, we found 4 SNPs in pre-SZmiRNAs and 18 SNPs in the expanded regions (100 bp each side) of pre-SZmiRNAs including one SNP (rs41283391) located 46 bp upstream of hsa-miR-195 pre-miRNA (see Additional file 3).

There were two publicly available GWA studies (CATIE and GAIN) for schizophrenia, neither of which has yet been successful in identifying significant genome-level markers [27]. Surprisingly, all of these SNPs except one (rs1700 in hsa-miR-198), were not included in either GWAS marker set. We found two potential regulatory SNPs in *GRM7*, one of the five core genes. These two SNPs were located on TFBS (V$AHRARNT_01, SNP rs62237229) and miRNA target site (hsa-miR-20b, SNP rs56173829). Both V$AHRARNT_01 and hsa-miR-20b were included in our FFLs. Our literature search revealed that these SNPs and sites have not been studied for schizophrenia. Further experimental verification is warranted.

### Online access of miRNAs and their targets in schizophrenia genes

We deposited all miRNAs complied in this study and their potential targets in schizophrenia genes into Schizophrenia Gene Resource (SZGR), a comprehensive online resource including genetic and biological data for schizophrenia genes [28]. SZGR deposits genetic data from all available sources including association studies, linkage scans, gene expression, literature, GO annotations, gene networks, pathways, and miRNAs and their target sites. Moreover, SZGR provides online tools for data browsing and searching, data integration, custom gene ranking, and graphical presentation.

## Discussion

### Potential regulatory networks in schizophrenia

We performed an exploratory miRNA-TF mediated regulatory network analysis, identifying some promising FFLs and mutual feedback loops in schizophrenia. In the converged network, we identified 5 core genes including *EGR3 *and hsa-miR-195 that likely play important regulatory roles. The network also includes some well-studied schizophrenia candidate genes (e.g., *BDNF*, *DRD2*, *GRIN1 *and *GAD1*). Although this investigation started from experimentally verified schizophrenia-related miRNAs, miRNA-TF-gene regulations, and a set of schizophrenia candidate genes prioritized by multiple lines of genetic evidence, most of the miRNA targets and TFBSs used in this study are putative and not error-free. At present, miRNAs have not been well tested for association with schizophrenia. The number of schizophrenia related miRNAs is expected to be greater than what we compiled. However, our analysis and subsequent permutation tests indicated that our regulatory network is nonrandom in the whole molecular network. The identified network modules provide potential targets for follow-up experimental verification, and provide important insights into the etiology of schizophrenia. We discuss some potential pathways below.

*EGR3 *encodes a zinc finger transcription factor and plays important roles in cellular growth, environmental stimuli, muscle-spindle development and neuronal development [29]. In neuronal development, *EGR3 *is required for normal terminal axon extension and branching, sympathetic target tissue innervation and function, and hippocampus-dependent learning and memory processing [30,31]. *EGR3 *indirectly modulates synaptic plasticity through its regulation of the *ARC *gene, a synaptic activity-induced effector molecule [32]. In developing neurons and epilepsy, *BDNF *is the endogenous signal that induces *EGR3 *expression via a PKC/MAPK-dependent pathway, and then EGR3 up-regulates the expression of *GABRA4 *by binding its promoter [33,34]. *EGR3 *is required in mediating the response to stress and novelty [35]. *EGR3 *has been reported to be associated with schizophrenia in both case-control and family-based studies and its expression has been shown to be decreased in schizophrenia patients [36,37]. Mice lacking *EGR3 *and schizophrenia patients display a similar decreased susceptibility to the side effects of antipsychotic medications [38]. These studies consistently suggest an important role for *EGR3 *in neuron activity and schizophrenia.

Moreover, *EGR3 *is a downstream gene of many signaling pathways including pathways triggered by *NGF*, *BDNF *and *NRG1 *[30,34,39,40], of which *BDNF *and *NRG1 *are schizophrenia susceptibility genes. Both *EGR1 *and *EGR2 *are induced by *BDNF *signaling in primary cortical neurons [41] and *EGR3 *has been proved to be a target gene of EGR1 [42]. EGR3 and EGR1 directly regulate the expression of *NGFR *(p75NTR) [43], a receptor of all neurotrophins, including NGF and BDNF. Interestingly, *NGFR *is involved in the regulation of axonal elongation [44] and *EGR3 *shares a similar function [30]. *EGR3 *is regulated by the calcium-responsive protein phosphatase calcineurin [45], which might be triggered by a calcium influx through NMDARs [46], whose activation also induces *EGR3 *mRNA expression [47]. *PPP3CC *(encoding calcineurin catalytic γ subunit) is located very close to *EGR3 *on chromosome 8 and was reported to be associated with schizophrenia [37,48]. Furthermore, the calcineurin/NFAT signaling pathway is required for neuronal development and axon growth, but it has little or no effect in neuron survival [49,50]. Interestingly, *EGR3 *is also required for normal axon extension and branching, but not for neuron survival [30]. Neurotrophins (NGF and BDNF) stimulate NFAT nuclear translocation and activation of NFAT-dependent transcription in cortical neurons [50]. It has been proposed that some unknown factors involved in calcineurin/NFAT signaling induce axon growth [49,50]. Based on these literature surveys and our miRNA-mediated regulatory network analysis, we propose that EGRs, especially EGR3, are key factors regulated by calcineurin/NFAT signaling in neuronal development. Moreover, in the immune system, NFAT directly transactivates *EGR3 *and *EGR2*, then activates the expression of *FasL *to trigger cell apoptosis [51].

**Figure 4:**
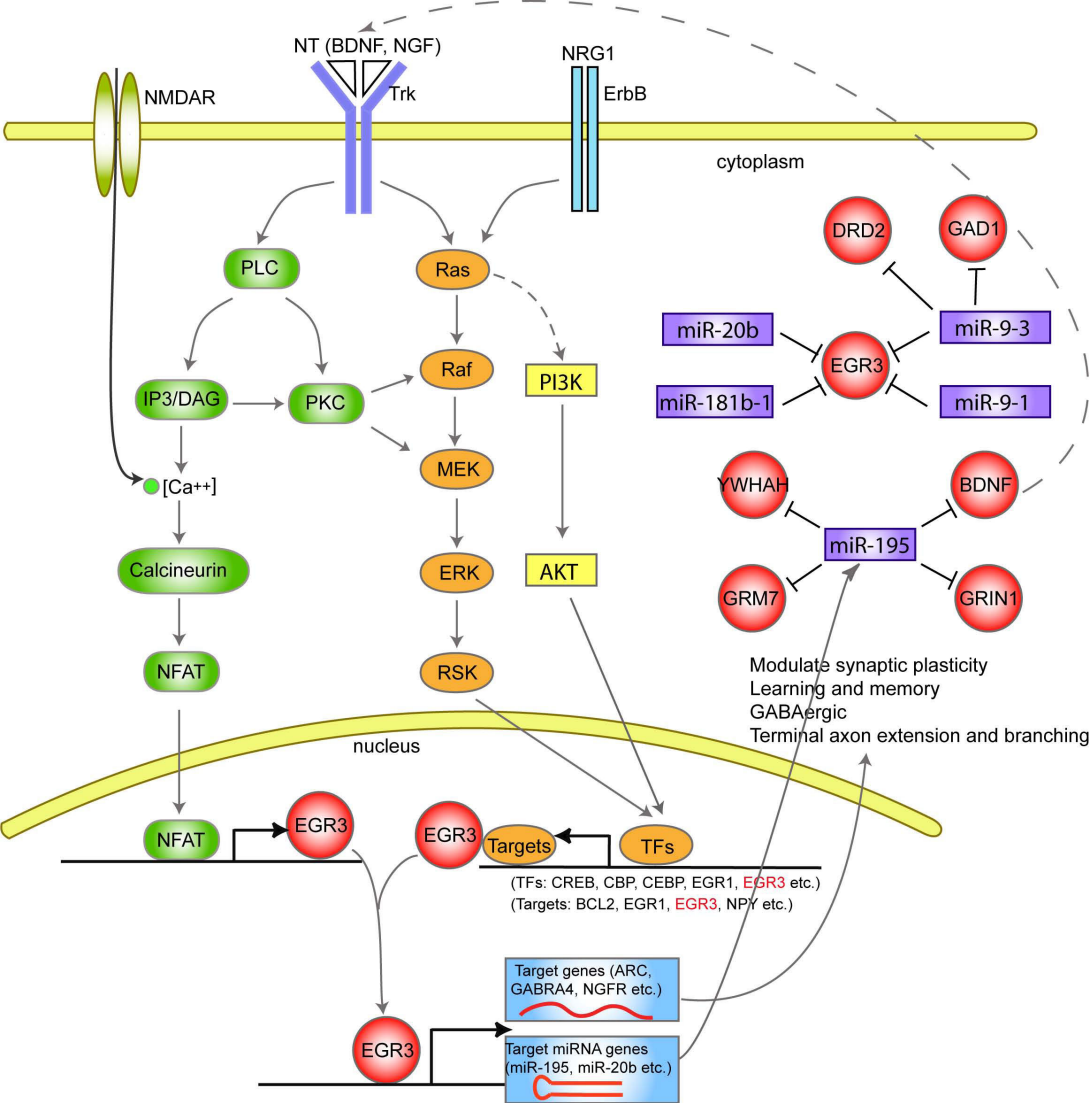
**Model of EGR3 and miRNAs involving in signaling pathways and regulatory networks in schizophrenia and nervous system**. Binding of neurotrophins (BDNF or NGF) to Trk receptors activates multiple signaling pathways, including Ras/MAPK cascade, PKC, PI3K/AKT, and IP3/calcium signaling [72,73]. Stimulation of NRG1 activates MAPK and PI3K/AKT pathways [74]. These signal transductions trigger activation of several TFs such as CREB, CBP, EGR1 and EGR3, which subsequently activate their target genes (e.g., *EGR3*, *BCL2 *and *NPY*). Calcium influx through NMDARs activates calcineurin/NFAT signaling pathway, then activates target genes such as *EGR3 *[46,49]. EGR3 regulates its target genes (e.g., *GABRA4*, *ARC *and *NGFR*) and miRNAs (e.g., hsa-miR-195 and hsa-miR-20b).

The above discussion led us to propose a model of the involvement of *EGR3 *and miRNAs in signaling pathways and regulatory networks within nervous system and schizophrenia (Figure 4). We inferred that *EGR *genes, especially *EGR3*, are downstream of *BDNF*, *NRG1*, and *NGF *via two pathways: MAPK-dependent signaling pathway and calcium-dependent calcineurin/NFAT signaling pathway. *EGR3 *expression is triggered by these two pathways after signal stimulation. Then, EGR3 activates its target protein-coding genes (e.g., *ARC*, *GABRA4 *and *NGFR*) and miRNAs (e.g., hsa-miR-195 and hsa-miR-20b). These target genes subsequently trigger downstream genes and pathways, inducing processes such as synaptic plasticity, axon extension, GABAergic and regulating expression of *BDNF *and *DRD2*.

### Hsa-miR-195 might prove a promising miRNA in schizophrenia and nervous system

Hsa-miR-195 is a core miRNA potentially targeted by 6 TFs and also targeting 6 SZGenes in our network. It was reported significantly down-regulated in the prefrontal cortex of schizophrenia patients [6]. So far, it has been the only miRNA whose regulation of schizophrenia genes has been verified by experimental evidence. It regulates *BDNF*, altering the expression of downstream GABAergic transcripts (*NPY*, *SST *and *PV*) in schizophrenia patients [10]. Note that *BDNF *also affects GABAergic system as a mediator of EGR3-induced *GABRA4 *regulation in developing neurons [34]. In our miRNA-mediated FFLs, EGR3 potentially regulates hsa-miR-195. Thus, hsa-miR-195, *BDNF *and *EGR3 *form a critical feedback regulatory loop (Figure 4). The predicted targets of hsa-miR-195 are enriched in neuron related pathways, such as axonal guidance signaling, reelin signaling in neurons, long term synaptic potentiation and Ephrin receptor signaling pathways (Table 4). In combination, the evidence above suggests that hsa-miR-195 might be a key miRNA in schizophrenia as well as nervous system.

### Potential utilities of FFLs and miRNA-TF composite feedback loops

FFL is an important regulatory motif and has been found in organisms such as *Escherichia coli*, yeast and human [52]. A traditional FFL is composed of two TFs and one target gene in gene expression regulation. Because miRNAs play key regulatory functions in gene expression, a FFL consisting of a TF, miRNA and a target gene is likely a powerful tool to investigate regulatory mechanisms of diseases at both the transcriptional and translational levels. It has been estimated that there are several thousands of human genes under this combinatory TF-miRNA regulation [14]. At present, only a few FFLs have been experimentally verified. Some examples include E2Fs-Myc-miR-17/20 [19], E2F-miR-106b/93/25-CDK inhibitors [53] and PKC-MAPK-miR-15a [54]. A miRNA-TF composite feedback loop is a direct regulation motif. Some experimental examples are ZEB1/SIP1 and the miR-200 family in embryologic development and Pitx3 and miR-133b in neuron development [17,18]. In our miRNA-mediated network in schizophrenia, we found an interesting miRNA-TF loop, the miR181-ESR1 loop. ESR1 is the only TF in our network whose gene has positive association result for schizophrenia [21]. Some SNPs in *ESR1 *were also found significantly associated with schizophrenia in our genotyping project (unpublished data). Additionally, Inoue et al. [55] suggested *EGR3 *being a target of ESR1 in breast cancer cells. Since *EGR3 *is a core gene in our miRNA-mediated schizophrenia network, this provides another link for *ESR1 *to schizophrenia.

## Conclusion

We compiled schizophrenia related miRNAs to predict SZmiRNA-TF-SZGene FFLs and found significantly more SZmiRNA-related FFLs in schizophrenia candidate genes. This is the first study of miRNA-TF regulatory networks in schizophrenia. We revealed that *EGR3 *and hsa-miR-195 are critical in the schizophrenia regulatory network. *EGR3 *is at the convergence of several signaling pathways, miRNA regulatory networks, adaptation to stress, and genetic susceptibility to schizophrenia. Although this study is exploratory, it provides an alternative and, perhaps, an effective approach for studying the regulatory mechanisms of genes involved in schizophrenia.

## Methods

### Schizophrenia, brain expressed, and non-brain expressed miRNAs

We compiled schizophrenia related miRNAs (SZmiRNAs) from three studies: (1) 16 miRNAs differentially expressed in prefrontal cortex of schizophrenia patients from controls [6]; (2) 2 miRNAs differentially expressed in postmortem cortical tissues of schizophrenia patients from controls [7]; and (3) 2 miRNAs in which 2 SNPs were found associated with schizophrenia in case/control samples [8]. For comparison, we compiled two control miRNA datasets: miRNAs expressed in brain (brain miRNAs) and non-brain tissues (non-brain miRNAs). Brain miRNAs were collected from miRNA microarray expression studies and a miRNA regulation survey study [7,56,57]. Non-brain miRNAs were collected from two large-scale miRNA expression atlas studies [58,59]. After we manually checked these miRNAs, we removed SZmiRNAs from brain miRNAs and, similarly, brain miRNAs from non-brain miRNAs to avoid redundancy. We obtained information of genomic locations, host genes and conservation among species of these miRNAs from miRBase (Release 11.0, genome assembly: NCBI36) [60].

### Schizophrenia candidate genes

We used a list of 160 schizophrenia candidate genes (SZGenes) prioritized by a multi-dimensional evidence-based prioritization approach [61]. These genes were selected based on integrative evidence from linkage, association, gene expression and literature search. Our follow up evaluation using independent GWAS *p*-values and gene expression features suggested these genes were promising [61]. Additionally, we compiled three other SZGene lists: (1) 270 genes having at least one positive association result in the SchizophreniaGene database (accessed in April 2008) [20], (2) 124 genes having at least two positive results in the SchizophreniaGene database, and (3) 75 SZGenes selected by a combined odds ratio method from association studies [62].

### Target prediction of miRNAs and transcription factors

Among many miRNA target prediction programs, TargetScan had the best performance based on two large scale miRNA induced protein synthesis studies [63,64]. We retrieved all the miRNA target prediction results from the TargetScan server (version 4.2, April 2008) [65] and then extracted the miRNA and target gene pairs by the corresponding miRNA lists (e.g., SZmiRNAs) and genes (e.g., SZGenes). Except one miRNA that is conserved only in primates (hsa-miR-198, Table 1), we required the miRNA target sites to be conserved in mammals. Although SZGenes tend to be longer [61], the length of the 3' untranslated regions (UTRs) in which target sites were predicted, was not found significantly different between the SZGenes and the other human genes (Wilcoxon test, *p *= 0.09). To examine whether SZmiRNAs have more miRNA targets in SZGenes than in non-SZGenes, we randomly selected the same number of genes from the human protein-coding genes and then counted the number of targets of each SZmiRNA for the random genes. We repeated this randomness analysis 10,000 times. Then, we used one sample t-test to test the significance.

miRNAs clustered in a genomic region are preferentially co-expressed and miRNAs in gene region are usually co-expressed with their host genes, presumably due to being part of the same transcription unit [58,59,66,67]. After comparing the miRNA cluster results of 3 kb, 5 kb, and 10 kb, we used a 5 kb maximum inter-miRNA distance as the miRNA cluster criteria, which is the same as in Xu and Wong [68]. Putative promoter regions of intergenic miRNAs were estimated up to several kb upstream from the miRNA precursors [13,69]. Here, we used 5 kb upstream of the host gene, miRNA precursor or miRNA cluster as the putative promoter region for miRNA in a genic region, intergenic region or miRNA cluster, respectively. Similar FFL results were found when we set a 1 kb promoter region (data not shown).

Because TFBS is always short, i.e., a 6-8 bp core sequence, prediction of TFBS in a single species may have a much higher false positive rate than that based on conservation across multiple species. We retrieved predicted TFBS information from the UCSC genome browser (hg18 genome assembly) and required TFBSs to be conserved among humans, mice and rats. To further reduce the false positive prediction, we used Z score of 2.33 as a cutoff for high quality TFBSs. A TFBS was considered associated with a target gene when it was in the gene's promoter region and its Z score was >2.33.

### Feed-forward loops (FFLs) and statistics tests

We analyzed FFLs for TFBSs, miRNAs and schizophrenia genes according to the procedure in Figure 1. Some TFBSs might overlap on their locations, could be bound by the same TF, or could be combined due to similar sequences. We manually merged those TFBSs to reduce redundancy.

Two methods were used to evaluate if the FFLs observed in the set of TFBSs, SZmiRNAs and SZGenes were significantly enriched from genome background. First, for the same SZGenes, we used Fisher's exact test to compare the observed FFLs from SZmiRNAs with those from brain miRNAs or non-brain miRNAs. Second, we ran randomization processes using the method in Shalgi et al [14]. In each run, we extracted the same number of random miRNA target pairs out of all predicted target pairs of the SZmiRNAs and identified TFBSs in the promoter of these random miRNA target genes, then calculated the number of FFLs. We repeated this 10,000 times, and set the *p*-value as the proportion of the random results that had no less than the number of FFLs observed in the set of SZmiRNAs and SZGenes.

### Network and pathway analysis

We used the Core Analysis tool in the Ingenuity Pathway Analysis (IPA) system [22] to analyze networks and pathways for a set of genes. We set *p*-value < 0.01 as the cutoff for enriched significant pathways identified by IPA. Networks were presented with Cytoscape software (version 2.6.0) [70]. In the miRNA-TF mediated network, when a schizophrenia gene was regulated by at least 3 TFs and at least 3 miRNAs, we defined it a core gene (hub gene). Similarly, when a miRNA regulated at least 3 SZGenes and was also regulated by at least 3 TFs, we defined it a core miRNA. Enriched GO terms for a set of genes were examined using the DAVID bioinformatics web server [71].

## Authors' contributions

AYG prepared the data, carried out the analysis and wrote the manuscript. JS participated in the statistics test and network analysis. PJ participated in data analysis and manuscript revision. ZZ conceived of the study, participated in its design and data interpretation, and contributed to the writing of the manuscript. All authors read and approved the final manuscript.

## Supplementary Material

Additional file 1 **Brain and non-brain expressed miRNAs**. This file lists the brain and non-brain expressed miRNAs. Click here for file

Additional file 2 **Supplementary tables and figures**. This file includes 3 supplementary tables and 3 supplementary figures. Supplementary table S1 shows the schizophrenia genes (SZGenes) targeted by more than one SZmiRNA and the number of SZGenes targeted by SZmiRNAs. Supplementary table S2 shows the SZmiRNA-TF mutual regulation loops found in this analysis. Supplementary table S3 shows the enriched GO terms in the predicted targets of 3 core miRNAs. Supplementary figure S1 depicts the comparison of the number of targets by SZmiRNAs in 160 schizophrenia genes and 160 randomly selected genes. Supplementary figure S2 depicts the distribution of the number of TFBSs in schizophrenia genes and SZmiRNAs. Supplementary figure S3 depicts the extracted subnetworks for core genes in miRNA-TF regulatory network. Click here for file

Additional file 3 **SNPs on TFBS, miRNA sites and miRNA genes**. This file includes the potential functional SNPs on TFBS, miRNA sites and miRNA genes. Click here for file

## References

[B1] Lang UE Puls I Muller DJ Strutz-Seebohm N Gallinat J Molecular mechanisms of schizophrenia Cell Physiol Biochem 2007 20 687 702 10.1159/000110430 17982252

[B2] Maycox PR Kelly F Taylor A Bates S Reid J Logendra R Barnes MR Larminie C Jones N Lennon M Analysis of gene expression in two large schizophrenia cohorts identifies multiple changes associated with nerve terminal function Mol Psychiatry 2009 14 1083 1094 10.1038/mp.2009.18 19255580

[B3] Bray NJ Gene expression in the etiology of schizophrenia Schizophr Bull 2008 34 412 418 10.1093/schbul/sbn013 18334509 PMC2632437

[B4] Sethupathy P Collins FS MicroRNA target site polymorphisms and human disease Trends Genet 2008 24 489 497 10.1016/j.tig.2008.07.004 18778868

[B5] Kuss AW Chen W MicroRNAs in brain function and disease Curr Neurol Neurosci Rep 2008 8 190 197 10.1007/s11910-008-0031-0 18541114

[B6] Perkins DO Jeffries CD Jarskog LF Thomson JM Woods K Newman MA Parker JS Jin J Hammond SM microRNA expression in the prefrontal cortex of individuals with schizophrenia and schizoaffective disorder Genome Biol 2007 8 R27 10.1186/gb-2007-8-2-r27 17326821 PMC1852419

[B7] Beveridge NJ Tooney PA Carroll AP Gardiner E Bowden N Scott RJ Tran N Dedova I Cairns MJ Dysregulation of miRNA 181b in the temporal cortex in schizophrenia Hum Mol Genet 2008 17 1156 1168 10.1093/hmg/ddn005 18184693

[B8] Hansen T Olsen L Lindow M Jakobsen KD Ullum H Jonsson E Andreassen OA Djurovic S Melle I Agartz I Brain expressed microRNAs implicated in schizophrenia etiology PLoS ONE 2007 2 e873 10.1371/journal.pone.0000873 17849003 PMC1964806

[B9] Stark KL Xu B Bagchi A Lai WS Liu H Hsu R Wan X Pavlidis P Mills AA Karayiorgou M Gogos JA Altered brain microRNA biogenesis contributes to phenotypic deficits in a 22q11-deletion mouse model Nat Genet 2008 40 751 760 10.1038/ng.138 18469815

[B10] Mellios N Huang HS Grigorenko A Rogaev E Akbarian S A set of differentially expressed miRNAs, including miR-30a-5p, act as post-transcriptional inhibitors of BDNF in prefrontal cortex Hum Mol Genet 2008 17 3030 3042 10.1093/hmg/ddn201 18632683 PMC2722882

[B11] Kocerha J Faghihi MA Lopez-Toledano MA Huang J Ramsey AJ Caron MG Sales N Willoughby D Elmen J Hansen HF MicroRNA-219 modulates NMDA receptor-mediated neurobehavioral dysfunction Proc Natl Acad Sci USA 2009 106 3507 3512 10.1073/pnas.0805854106 19196972 PMC2651305

[B12] Coyle JT MicroRNAs suggest a new mechanism for altered brain gene expression in schizophrenia Proc Natl Acad Sci USA 2009 106 2975 2976 10.1073/pnas.0813321106 19251669 PMC2651256

[B13] Saini HK Griffiths-Jones S Enright AJ Genomic analysis of human microRNA transcripts Proc Natl Acad Sci USA 2007 104 17719 17724 10.1073/pnas.0703890104 17965236 PMC2077053

[B14] Shalgi R Lieber D Oren M Pilpel Y Global and local architecture of the mammalian microRNA-transcription factor regulatory network PLoS Comput Biol 2007 3 e131 10.1371/journal.pcbi.0030131 17630826 PMC1914371

[B15] Tsang J Zhu J van Oudenaarden A MicroRNA-mediated feedback and feedforward loops are recurrent network motifs in mammals Mol Cell 2007 26 753 767 10.1016/j.molcel.2007.05.018 17560377 PMC2072999

[B16] Martinez NJ Ow MC Barrasa MI Hammell M Sequerra R Doucette-Stamm L Roth FP Ambros VR Walhout AJ A C. elegans genome-scale microRNA network contains composite feedback motifs with high flux capacity Genes Dev 2008 22 2535 2549 10.1101/gad.1678608 18794350 PMC2546694

[B17] Bracken CP Gregory PA Kolesnikoff N Bert AG Wang J Shannon MF Goodall GJ A double-negative feedback loop between ZEB1-SIP1 and the microRNA-200 family regulates epithelial-mesenchymal transition Cancer Res 2008 68 7846 7854 10.1158/0008-5472.CAN-08-1942 18829540

[B18] Kim J Inoue K Ishii J Vanti WB Voronov SV Murchison E Hannon G Abeliovich A A MicroRNA feedback circuit in midbrain dopamine neurons Science 2007 317 1220 1224 10.1126/science.1140481 17761882 PMC2782470

[B19] Sylvestre Y De Guire V Querido E Mukhopadhyay UK Bourdeau V Major F Ferbeyre G Chartrand P An E2F/miR-20a autoregulatory feedback loop J Biol Chem 2007 282 2135 2143 10.1074/jbc.M608939200 17135249

[B20] Allen NC Bagade S McQueen MB Ioannidis JP Kavvoura FK Khoury MJ Tanzi RE Bertram L Systematic meta-analyses and field synopsis of genetic association studies in schizophrenia: the SzGene database Nat Genet 2008 40 827 834 10.1038/ng.171 18583979

[B21] Weickert CS Miranda-Angulo AL Wong J Perlman WR Ward SE Radhakrishna V Straub RE Weinberger DR Kleinman JE Variants in the estrogen receptor alpha gene and its mRNA contribute to risk for schizophrenia Hum Mol Genet 2008 17 2293 2309 10.1093/hmg/ddn130 18424448 PMC2465798

[B22] Ingenuity Systems http://www.ingenuity.com/

[B23] Negishi M Oinuma I Katoh H Plexins: axon guidance and signal transduction Cell Mol Life Sci 2005 62 1363 1371 10.1007/s00018-005-5018-2 15818466 PMC11139078

[B24] Klein R Bidirectional modulation of synaptic functions by Eph/ephrin signaling Nat Neurosci 2009 12 15 20 10.1038/nn.2231 19029886

[B25] De Gobbi M Viprakasit V Hughes JR Fisher C Buckle VJ Ayyub H Gibbons RJ Vernimmen D Yoshinaga Y de Jong P A regulatory SNP causes a human genetic disease by creating a new transcriptional promoter Science 2006 312 1215 1217 10.1126/science.1126431 16728641

[B26] Tokuhiro S Yamada R Chang X Suzuki A Kochi Y Sawada T Suzuki M Nagasaki M Ohtsuki M Ono M An intronic SNP in a RUNX1 binding site of SLC22A4, encoding an organic cation transporter, is associated with rheumatoid arthritis Nat Genet 2003 35 341 348 10.1038/ng1267 14608356

[B27] Sullivan PF Lin D Tzeng JY Oord E van den Perkins D Stroup TS Wagner M Lee S Wright FA Zou F Genomewide association for schizophrenia in the CATIE study: results of stage 1 Mol Psychiatry 2008 13 570 584 10.1038/mp.2008.25 18347602 PMC3910086

[B28] Jia P Sun J Guo A Zhao Z SZGR: a comprehensive schizophrenia gene resource Mol Psychiatry 2010 in press 10.1038/mp.2009.93 PMC2861797 20424623

[B29] O'Donovan KJ Tourtellotte WG Millbrandt J Baraban JM The EGR family of transcription-regulatory factors: progress at the interface of molecular and systems neuroscience Trends Neurosci 1999 22 167 173 10.1016/S0166-2236(98)01343-5 10203854

[B30] Eldredge LC Gao XM Quach DH Li L Han X Lomasney J Tourtellotte WG Abnormal sympathetic nervous system development and physiological dysautonomia in Egr3-deficient mice Development 2008 135 2949 2957 10.1242/dev.023960 18653557 PMC2613541

[B31] Li L Yun SH Keblesh J Trommer BL Xiong H Radulovic J Tourtellotte WG Egr3, a synaptic activity regulated transcription factor that is essential for learning and memory Mol Cell Neurosci 2007 35 76 88 10.1016/j.mcn.2007.02.004 17350282 PMC2683345

[B32] Li L Carter J Gao X Whitehead J Tourtellotte WG The neuroplasticity-associated arc gene is a direct transcriptional target of early growth response (Egr) transcription factors Mol Cell Biol 2005 25 10286 10300 10.1128/MCB.25.23.10286-10300.2005 16287845 PMC1291244

[B33] Roberts DS Raol YH Bandyopadhyay S Lund IV Budreck EC Passini MA Wolfe JH Brooks-Kayal AR Russek SJ Egr3 stimulation of GABRA4 promoter activity as a mechanism for seizure-induced up-regulation of GABA(A) receptor alpha4 subunit expression Proc Natl Acad Sci USA 2005 102 11894 11899 10.1073/pnas.0501434102 16091474 PMC1187961

[B34] Roberts DS Hu Y Lund IV Brooks-Kayal AR Russek SJ Brain-derived neurotrophic factor (BDNF)-induced synthesis of early growth response factor 3 (Egr3) controls the levels of type A GABA receptor alpha 4 subunits in hippocampal neurons J Biol Chem 2006 281 29431 29435 10.1074/jbc.C600167200 16901909

[B35] Gallitano-Mendel A Izumi Y Tokuda K Zorumski CF Howell MP Muglia LJ Wozniak DF Milbrandt J The immediate early gene early growth response gene 3 mediates adaptation to stress and novelty Neuroscience 2007 148 633 643 10.1016/j.neuroscience.2007.05.050 17692471 PMC2597331

[B36] Mexal S Frank M Berger R Adams CE Ross RG Freedman R Leonard S Differential modulation of gene expression in the NMDA postsynaptic density of schizophrenic and control smokers Brain Res Mol Brain Res 2005 139 317 332 10.1016/j.molbrainres.2005.06.006 16122832

[B37] Yamada K Gerber DJ Iwayama Y Ohnishi T Ohba H Toyota T Aruga J Minabe Y Tonegawa S Yoshikawa T Genetic analysis of the calcineurin pathway identifies members of the EGR gene family, specifically EGR3, as potential susceptibility candidates in schizophrenia Proc Natl Acad Sci USA 2007 104 2815 2820 10.1073/pnas.0610765104 17360599 PMC1815264

[B38] Gallitano-Mendel A Wozniak DF Pehek EA Milbrandt J Mice lacking the immediate early gene Egr3 respond to the anti-aggressive effects of clozapine yet are relatively resistant to its sedating effects Neuropsychopharmacology 2008 33 1266 1275 10.1038/sj.npp.1301505 17637609 PMC4621766

[B39] Santos AR Duarte CB Validation of internal control genes for expression studies: effects of the neurotrophin BDNF on hippocampal neurons J Neurosci Res 2008 86 3684 3692 10.1002/jnr.21796 18655199

[B40] Jacobson C Duggan D Fischbach G Neuregulin induces the expression of transcription factors and myosin heavy chains typical of muscle spindles in cultured human muscle Proc Natl Acad Sci USA 2004 101 12218 12223 10.1073/pnas.0404240101 15302938 PMC514402

[B41] Calella AM Nerlov C Lopez RG Sciarretta C von Bohlen Halbach O Bereshchenko O Minichiello L Neurotrophin/Trk receptor signaling mediates C/EBPalpha, -beta and NeuroD recruitment to immediate-early gene promoters in neuronal cells and requires C/EBPs to induce immediate-early gene transcription Neural Develop 2007 2 4 10.1186/1749-8104-2-4 PMC1796876 17254333

[B42] Ehrengruber MU Muhlebach SG Sohrman S Leutenegger CM Lester HA Davidson N Modulation of early growth response (EGR) transcription factor-dependent gene expression by using recombinant adenovirus Gene 2000 258 63 69 10.1016/S0378-1119(00)00445-5 11111043

[B43] Gao X Daugherty RL Tourtellotte WG Regulation of low affinity neurotrophin receptor (p75(NTR)) by early growth response (Egr) transcriptional regulators Mol Cell Neurosci 2007 36 501 514 10.1016/j.mcn.2007.08.013 17916431 PMC2098703

[B44] Dechant G Barde YA The neurotrophin receptor p75(NTR): novel functions and implications for diseases of the nervous system Nat Neurosci 2002 5 1131 1136 10.1038/nn1102-1131 12404007

[B45] Mittelstadt PR Ashwell JD Cyclosporin A-sensitive transcription factor Egr-3 regulates Fas ligand expression Mol Cell Biol 1998 18 3744 3751 9632757 10.1128/mcb.18.7.3744 PMC108957

[B46] Sheng M Kim MJ Postsynaptic signaling and plasticity mechanisms Science 2002 298 776 780 10.1126/science.1075333 12399578

[B47] Yamagata K Kaufmann WE Lanahan A Papapavlou M Barnes CA Andreasson KI Worley PF Egr3/Pilot, a zinc finger transcription factor, is rapidly regulated by activity in brain neurons and colocalizes with Egr1/zif268 Learn Mem 1994 1 140 152 10467592

[B48] Gerber DJ Hall D Miyakawa T Demars S Gogos JA Karayiorgou M Tonegawa S Evidence for association of schizophrenia with genetic variation in the 8p21.3 gene, PPP3CC, encoding the calcineurin gamma subunit Proc Natl Acad Sci USA 2003 100 8993 8998 10.1073/pnas.1432927100 12851458 PMC166426

[B49] Nguyen T Di Giovanni S NFAT signaling in neural development and axon growth Int J Dev Neurosci 2008 26 141 145 10.1016/j.ijdevneu.2007.10.004 18093786 PMC2267928

[B50] Graef IA Wang F Charron F Chen L Neilson J Tessier-Lavigne M Crabtree GR Neurotrophins and netrins require calcineurin/NFAT signaling to stimulate outgrowth of embryonic axons Cell 2003 113 657 670 10.1016/S0092-8674(03)00390-8 12787506

[B51] Rengarajan J Mittelstadt PR Mages HW Gerth AJ Kroczek RA Ashwell JD Glimcher LH Sequential involvement of NFAT and Egr transcription factors in FasL regulation Immunity 2000 12 293 300 10.1016/S1074-7613(00)80182-X 10755616

[B52] Mangan S Alon U Structure and function of the feed-forward loop network motif Proc Natl Acad Sci USA 2003 100 11980 11985 10.1073/pnas.2133841100 14530388 PMC218699

[B53] Brosh R Shalgi R Liran A Landan G Korotayev K Nguyen GH Enerly E Johnsen H Buganim Y Solomon H p53-Repressed miRNAs are involved with E2F in a feed-forward loop promoting proliferation Mol Syst Biol 2008 4 229 10.1038/msb.2008.65 19034270 PMC2600669

[B54] Cohen EE Zhu H Lingen MW Martin LE Kuo WL Choi EA Kocherginsky M Parker JS Chung CH Rosner MR A feed-forward loop involving protein kinase Calpha and microRNAs regulates tumor cell cycle Cancer Res 2009 69 65 74 10.1158/0008-5472.CAN-08-0377 19117988 PMC2746005

[B55] Inoue A Omoto Y Yamaguchi Y Kiyama R Hayashi SI Transcription factor EGR3 is involved in the estrogen-signaling pathway in breast cancer cells J Mol Endocrinol 2004 32 649 661 10.1677/jme.0.0320649 15171706

[B56] Burmistrova OA Goltsov AY Abramova LI Kaleda VG Orlova VA Rogaev EI MicroRNA in schizophrenia: genetic and expression analysis of miR-130b (22q11) Biochemistry (Mosc) 2007 72 578 582 10.1134/S0006297907050161 17573714

[B57] Zhang R Su B MicroRNA regulation and the variability of human cortical gene expression Nucleic Acids Res 2008 36 4621 4628 10.1093/nar/gkn431 18617573 PMC2504318

[B58] Landgraf P Rusu M Sheridan R Sewer A Iovino N Aravin A Pfeffer S Rice A Kamphorst AO Landthaler M A mammalian microRNA expression atlas based on small RNA library sequencing Cell 2007 129 1401 1414 10.1016/j.cell.2007.04.040 17604727 PMC2681231

[B59] Liang Y Ridzon D Wong L Chen C Characterization of microRNA expression profiles in normal human tissues BMC Genomics 2007 8 166 10.1186/1471-2164-8-166 17565689 PMC1904203

[B60] Griffiths-Jones S Saini HK van Dongen S Enright AJ miRBase: tools for microRNA genomics Nucleic Acids Res 2008 36 D154 158 10.1093/nar/gkm952 17991681 PMC2238936

[B61] Sun J Jia P Fanous AH Webb BT Oord EJCG van den Chen X Bukszar J Kendler KS Zhao Z A multi-dimensional evidence-based candidate gene prioritization approach for complex diseases - schizophrenia as a case Bioinformatics 2009 25 2595 2602 10.1093/bioinformatics/btp428 19602527 PMC2752609

[B62] Sun J Kuo PH Riley BP Kendler KS Zhao Z Candidate genes for schizophrenia: a survey of association studies and gene ranking Am J Med Genet B Neuropsychiatr Genet 2008 147B 1173 1181 10.1002/ajmg.b.30743 18361404

[B63] Selbach M Schwanhausser B Thierfelder N Fang Z Khanin R Rajewsky N Widespread changes in protein synthesis induced by microRNAs Nature 2008 455 58 63 10.1038/nature07228 18668040

[B64] Baek D Villen J Shin C Camargo FD Gygi SP Bartel DP The impact of microRNAs on protein output Nature 2008 455 64 71 10.1038/nature07242 18668037 PMC2745094

[B65] TargetScan: Prediction of microRNA targets http://www.targetscan.org/vert_42/

[B66] Baskerville S Bartel DP Microarray profiling of microRNAs reveals frequent coexpression with neighboring miRNAs and host genes Rna 2005 11 241 247 10.1261/rna.7240905 15701730 PMC1370713

[B67] Rodriguez A Griffiths-Jones S Ashurst JL Bradley A Identification of mammalian microRNA host genes and transcription units Genome Res 2004 14 1902 1910 10.1101/gr.2722704 15364901 PMC524413

[B68] Xu J Wong C A computational screen for mouse signaling pathways targeted by microRNA clusters Rna 2008 14 1276 1283 10.1261/rna.997708 18511500 PMC2441985

[B69] Zhou X Ruan J Wang G Zhang W Characterization and identification of microRNA core promoters in four model species PLoS Comput Biol 2007 3 e37 10.1371/journal.pcbi.0030037 17352530 PMC1817659

[B70] Shannon P Markiel A Ozier O Baliga NS Wang JT Ramage D Amin N Schwikowski B Ideker T Cytoscape: a software environment for integrated models of biomolecular interaction networks Genome Res 2003 13 2498 2504 10.1101/gr.1239303 14597658 PMC403769

[B71] DAVID Bioinformatics Resources http://david.abcc.ncifcrf.gov/

[B72] Hu Y Russek SJ BDNF and the diseased nervous system: a delicate balance between adaptive and pathological processes of gene regulation J Neurochem 2008 105 1 17 10.1111/j.1471-4159.2008.05237.x 18208542

[B73] Patapoutian A Reichardt LF Trk receptors: mediators of neurotrophin action Curr Opin Neurobiol 2001 11 272 280 10.1016/S0959-4388(00)00208-7 11399424

[B74] Mei L Xiong WC Neuregulin 1 in neural development, synaptic plasticity and schizophrenia Nat Rev Neurosci 2008 9 437 452 10.1038/nrn2392 18478032 PMC2682371

