# Supplementary material for: A Novel microRNA and transcription factor mediated regulatory network in schizophrenia
Source: BMC Syst Biol. 2010 Feb 15;4:10. doi: 10.1186/1752-0509-4-10 (PMC2834616; doi:10.1186/1752-0509-4-10)

Supplementary Tables

Table S1 Schizophrenia genes (SZGenes) targeted by more than one SZmiRNA and the number of SZGenes targeted by SZmiRNAs

**SZGene**	**No. of miRNAs that target SZGene**	**miRNA**	**No. of SZGenes targeted by miRNA**
EGR3	15	hsa-miR-198	23
DPYSL2	13	hsa-miR-30e	14
CNR1	11	hsa-miR-30d	14
SLC1A2	10	hsa-miR-30b	14
STX1A	8	hsa-miR-30a	14
NR4A2	8	hsa-miR-195	11
GRM7	8	hsa-miR-20b	8
SYN2	7	hsa-miR-181b-2	8
NTNG1	7	hsa-miR-181b-1	8
BDNF	7	hsa-miR-106b	8
PLXNA2	6	hsa-miR-9-3	7
NEUROG1	6	hsa-miR-9-2	7
IL1A	6	hsa-miR-9-1	7
MAGI2	5	hsa-miR-26b	6
GAD1	5	hsa-let-7g	6
ERBB4	5	hsa-miR-92b	5
TSNAX	4	hsa-miR-92a-2	5
PIP5K2A	4	hsa-miR-92a-1	5
GRM3	4	hsa-miR-29c	5
GRIA4	4	hsa-miR-29b-2	5
GABRB2	4	hsa-miR-29b-1	5
C18orf1	4	hsa-miR-29a	5
ARVCF	4	hsa-miR-24-2	4
HTR4	3	hsa-miR-24-1	4
GCLM	3	hsa-miR-206	3
GABRA1	3	hsa-miR-7-3	2
DRD2	3	hsa-miR-7-2	2
ST8SIA2	2	hsa-miR-7-1	2
SLC6A4	2	hsa-miR-212	2
RANBP5	2		
NPAS3	2		
MTHFR	2		
MCHR1	2		
MAGI1	2		
GRID1	2		

Table S2 SZmiRNA-TF mutual regulation loops

No.	SZmiRNA	TF
1	hsa-miR-181b-1	ESR1
2	hsa-miR-195	MYB
3	hsa-miR-20b	EGR3
4	hsa-miR-20b	MYCN
5	hsa-miR-20b	TAL1
6	hsa-miR-24-2	MAFG
7	hsa-miR-26b	EP300
8	hsa-miR-29b-2	MAFG
9	hsa-miR-29c	MAFG
10	hsa-miR-9-1	POU2F2
11	hsa-miR-9-1	POU3F2
12	hsa-miR-92a-1	CREB1
13	hsa-miR-92a-1	TFAP2A
14	hsa-miR-92b	RFX1

Five pairs (No. 3, 5, 8, 9, 10) also appeared in the FFLs.

Table S3 Enriched GO terms in the predicted targets of 3 core miRNAs

GO term	*p*-value
**hsa-miR-195**	
GO:0006464~protein modification process	7.69×10^{-12}
GO:0000074~regulation of progression through cell cycle	1.41×10^{-8}
GO:0004672~protein kinase activity	3.12×10^{-6}
GO:0019219~regulation of nucleobase, nucleoside, nucleotide and nucleic acid metabolic process	5.99×10^{-5}
GO:0009952~anterior/posterior pattern formation	6.95×10^{-5}
GO:0030182~neuron differentiation	1.30×10^{-4}
GO:0045449~regulation of transcription	1.62×10^{-4}
GO:0006355~regulation of transcription, DNA-dependent	2.26×10^{-4}
GO:0022008~neurogenesis	2.42×10^{-4}
GO:0006351~transcription, DNA-dependent	2.58×10^{-4}
GO:0032774~RNA biosynthetic process	2.76×10^{-4}
GO:0005875~microtubule associated complex	3.20×10^{-4}
GO:0043549~regulation of kinase activity	3.98×10^{-4}
GO:0016055~Wnt receptor signaling pathway	4.12×10^{-4}
GO:0016310~phosphorylation	4.52×10^{-4}
GO:0043632~modification-dependent macromolecule catabolic process	4.82×10^{-4}
GO:0005871~kinesin complex	5.06×10^{-4}
GO:0048666~neuron development	5.81×10^{-4}
GO:0044257~cellular protein catabolic process	6.18×10^{-4}
GO:0015630~microtubule cytoskeleton	6.49×10^{-4}
GO:0006897~endocytosis	7.41×10^{-4}
GO:0005874~microtubule	8.45×10^{-4}
**hsa-miR-20b**	
GO:0019219~regulation of nucleobase, nucleoside, nucleotide and nucleic acid metabolic process	2.64×10^{-18}
GO:0045449~regulation of transcription	1.51×10^{-17}
GO:0006351~transcription, DNA-dependent	5.07×10^{-16}
GO:0032774~RNA biosynthetic process	5.43×10^{-16}
GO:0006355~regulation of transcription, DNA-dependent	6.95×10^{-16}
GO:0005634~nucleus	1.61×10^{-15}
GO:0006357~regulation of transcription from RNA polymerase II promoter	9.25×10^{-12}
GO:0006464~protein modification process	2.55×10^{-10}
GO:0045893~positive regulation of transcription, DNA-dependent	4.81×10^{-9}
GO:0045935~positive regulation of nucleobase, nucleoside, nucleotide and nucleic acid metabolic process	2.01×10^{-8}
GO:0045941~positive regulation of transcription	9.24×10^{-8}
GO:0031325~positive regulation of cellular metabolic process	2.23×10^{-7}
GO:0004672~protein kinase activity	2.77×10^{-7}
GO:0007264~small GTPase mediated signal transduction	5.59×10^{-7}
GO:0043231~intracellular membrane-bound organelle	6.56×10^{-7}
GO:0043229~intracellular organelle	8.78×10^{-7}
GO:0004709~MAP kinase kinase kinase activity	2.54×10^{-6}
GO:0019787~small conjugating protein ligase activity	1.34×10^{-5}
GO:0000074~regulation of progression through cell cycle	1.37×10^{-5}
GO:0031324~negative regulation of cellular metabolic process	3.24×10^{-5}
GO:0000902~cell morphogenesis	4.51×10^{-5}
GO:0045892~negative regulation of transcription, DNA-dependent	4.85×10^{-5}
GO:0016481~negative regulation of transcription	6.77×10^{-5}
GO:0007167~enzyme linked receptor protein signaling pathway	7.38×10^{-5}
GO:0008270~zinc ion binding	9.71×10^{-5}
GO:0045934~negative regulation of nucleobase, nucleoside, nucleotide and nucleic acid metabolic process	1.25×10^{-4}

GO:0022008~neurogenesis	1.32×10^{-4}
GO:0001558~regulation of cell growth	1.38×10^{-4}
GO:0030182~neuron differentiation	1.68×10^{-4}
GO:0016310~phosphorylation	2.22×10^{-4}
GO:0045786~negative regulation of progression through cell cycle	2.63×10^{-4}
GO:0051329~interphase of mitotic cell cycle	2.82×10^{-4}
GO:0030308~negative regulation of cell growth	2.94×10^{-4}
GO:0008361~regulation of cell size	3.37×10^{-4}
GO:0043549~regulation of kinase activity	4.98×10^{-4}
GO:0051325~interphase	5.09×10^{-4}
GO:0044451~nucleoplasm part	5.82×10^{-4}
GO:0051056~regulation of small GTPase mediated signal transduction	8.10×10^{-4}
GO:0006325~establishment and/or maintenance of chromatin architecture	8.39×10^{-4}
GO:0031981~nuclear lumen	8.67×10^{-4}
GO:0005654~nucleoplasm	9.64×10^{-4}
**hsa-miR-9-3**	
GO:0019219~regulation of nucleobase, nucleoside, nucleotide and nucleic acid metabolic process	7.73×10^{-9}
GO:0006355~regulation of transcription, DNA-dependent	1.52×10^{-8}
GO:0045449~regulation of transcription	2.11×10^{-8}
GO:0006351~transcription, DNA-dependent	4.21×10^{-8}
GO:0032774~RNA biosynthetic process	4.64×10^{-8}
GO:0005634~nucleus	2.33×10^{-7}
GO:0006464~protein modification process	1.03×10^{-6}
GO:0030054~cell junction	1.24×10^{-6}
GO:0004672~protein kinase activity	2.66×10^{-5}
GO:0005737~cytoplasm	5.42×10^{-5}
GO:0016481~negative regulation of transcription	9.54×10^{-5}
GO:0043231~intracellular membrane-bound organelle	1.06×10^{-4}
GO:0006357~regulation of transcription from RNA polymerase II promoter	1.16×10^{-4}
GO:0008270~zinc ion binding	2.33×10^{-4}
GO:0045935~positive regulation of nucleobase, nucleoside, nucleotide and nucleic acid metabolic process	3.03×10^{-4}
GO:0000902~cell morphogenesis	3.15×10^{-4}
GO:0031324~negative regulation of cellular metabolic process	3.52×10^{-4}
GO:0045941~positive regulation of transcription	3.99×10^{-4}
GO:0045934~negative regulation of nucleobase, nucleoside, nucleotide and nucleic acid metabolic process	4.41×10^{-4}
GO:0043229~intracellular organelle	4.78×10^{-4}
GO:0005794~Golgi apparatus	5.08×10^{-4}
GO:0030135~coated vesicle	6.62×10^{-4}
GO:0045893~positive regulation of transcription, DNA-dependent	8.65×10^{-4}

Supplementary Figures

Figure S1 Comparison of number of targets by SZmiRNAs in 160 schizophrenia genes and 160 random genes. The randomization of 160 genes repeated 10,000 times and the standard deviation is shown on the white bars. Significance between the number of targets in 160 SZGenes and 160 random genes was tested by t-test. Except for hsa-miR-7-1, hsa-miR-7-2, and hsa-miR-7-3, all other miRNAs had significantly more targets in SZGenes than in random genes.

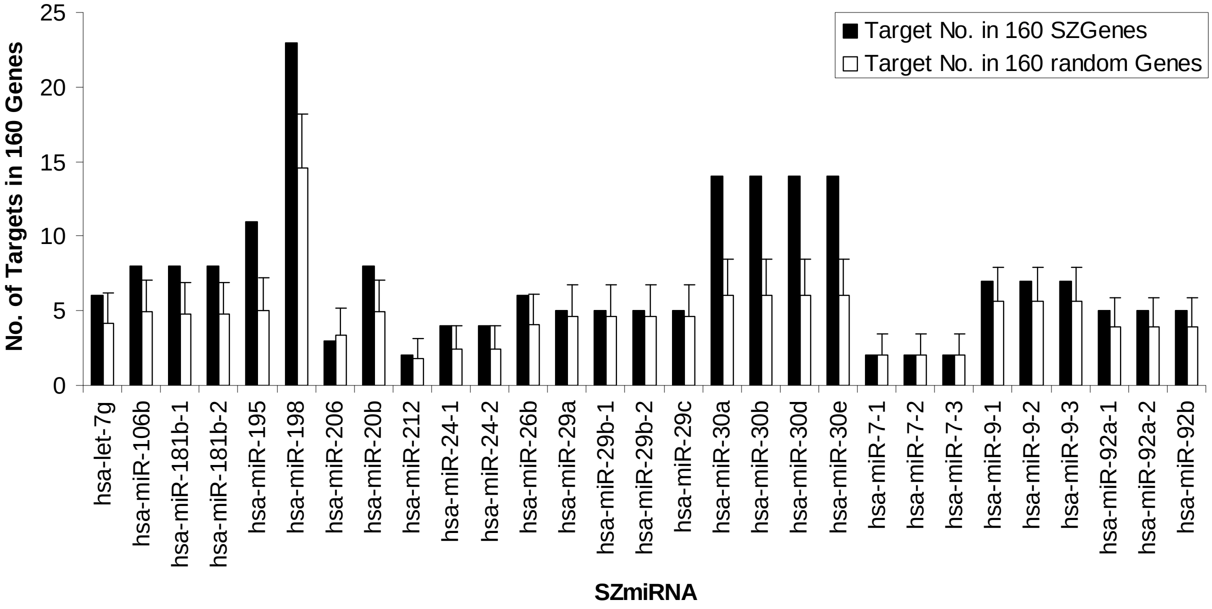

Figure S2 Distribution of the number of TFBSs in schizophrenia genes and SZmiRNAs.

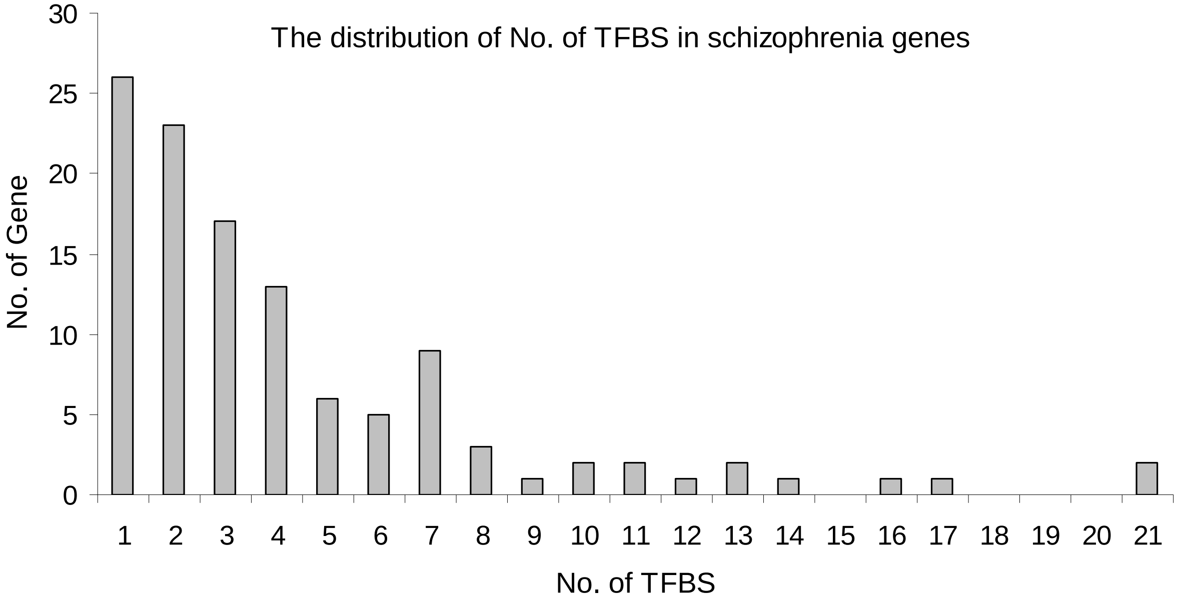

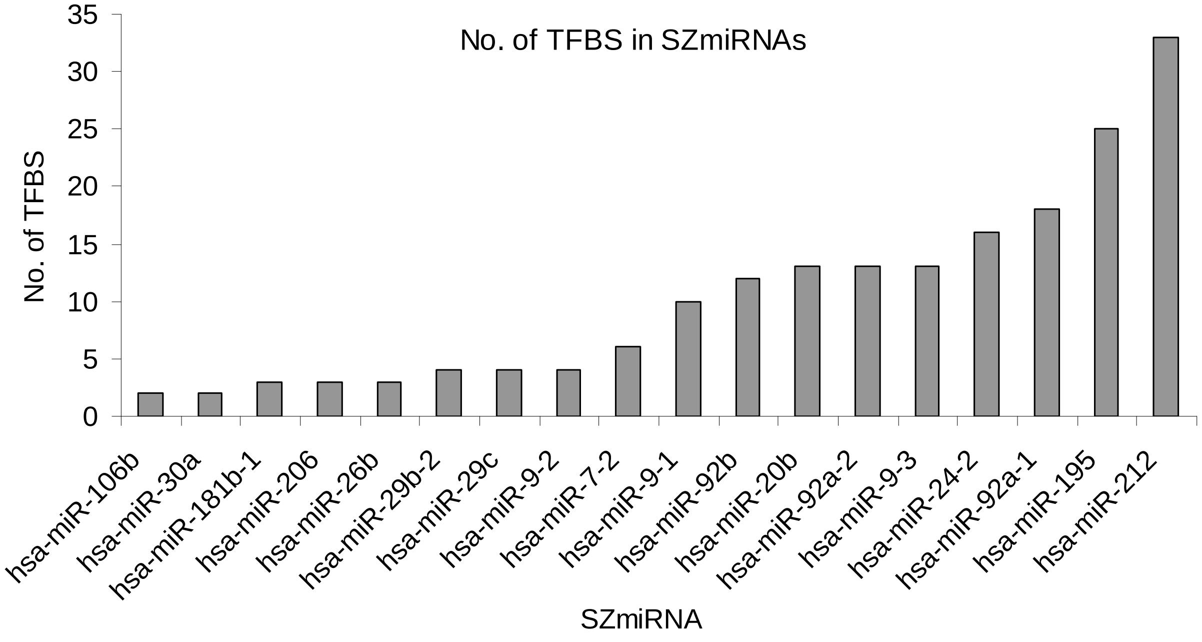

Figure S3 Extracted subnetworks for core genes in miRNA-TF regulatory network. In Fig S3B, has-miR-195 is potentially regulated by 6 TFs including EGR3.

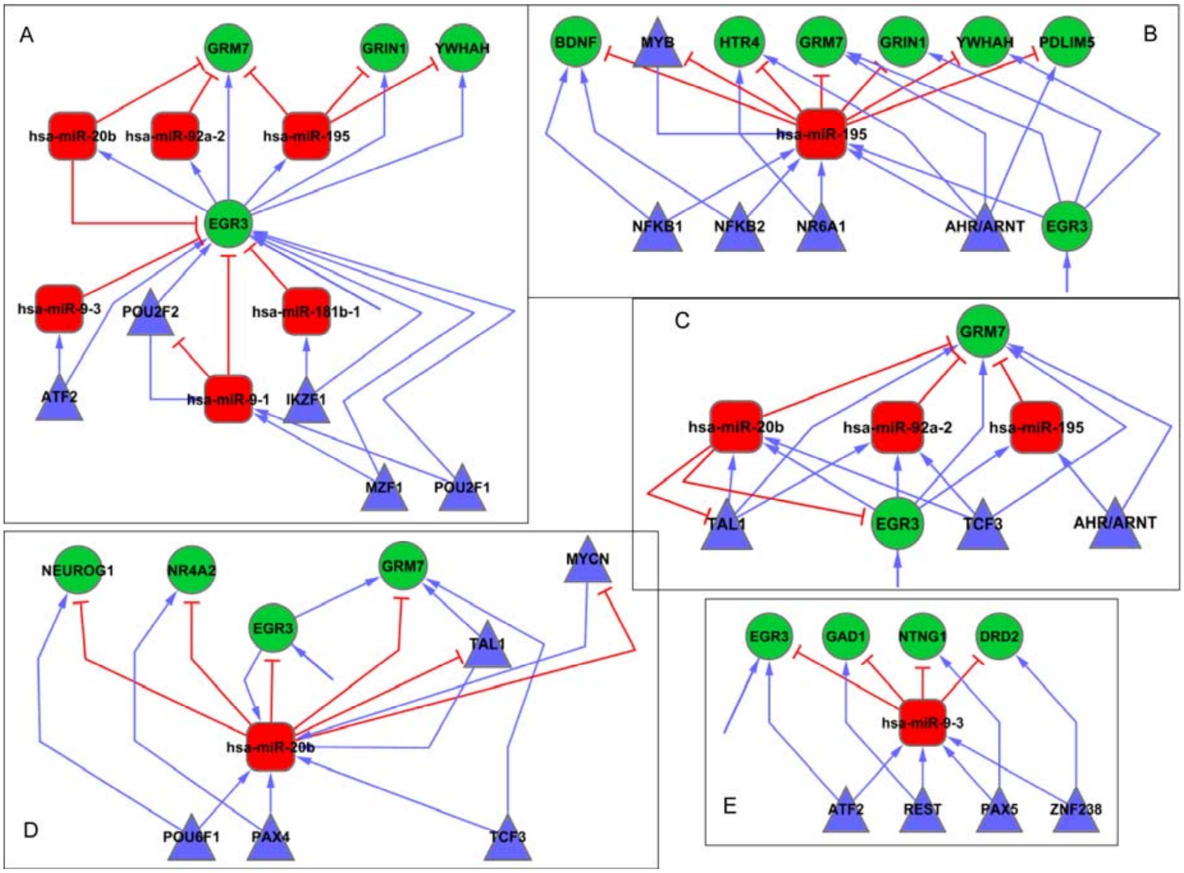

Supplement: Additional file 2 — Supplementary tables and figures. This file includes 3 supplementary tables and 3 supplementary figures. Supplementary table S1 shows the schizophrenia genes (SZGenes) targeted by more than one SZmiRNA and the number of SZGenes targeted by SZmiRNAs. Supplementary table S2 shows the SZmiRNA-TF mutual regulation loops found in this analysis. Supplementary table S3 shows the enriched GO terms in the predicted targets of 3 core miRNAs. Supplementary figure S1 depicts the comparison of the number of targets by SZmiRNAs in 160 schizophrenia genes and 160 randomly selected genes. Supplementary figure S2 depicts the distribution of the number of TFBSs in schizophrenia genes and SZmiRNAs. Supplementary figure S3 depicts the extracted subnetworks for core genes in miRNA-TF regulatory network. [file 1752-0509-4-10-S2.PDF]
